# Impact of a child with congenital anomalies on parents (ICCAP) questionnaire; a psychometric analysis

**DOI:** 10.1186/1477-7525-6-102

**Published:** 2008-11-23

**Authors:** Petra Mazer, Saskia J Gischler, Hans M Koot, Dick Tibboel, Monique van Dijk, Hugo J Duivenvoorden

**Affiliations:** 1 Intensive Care, Department of Pediatric Surgery, Erasmus MC – Sophia Children's Hospital, Erasmus University Medical Center Rotterdam, the Netherlands; 2 Department of Developmental Psychology, Vrije Universiteit Amsterdam, the Netherlands; 3 Department of Medical Psychology and Psychotherapy, Erasmus University Medical Center Rotterdam, the Netherlands

## Abstract

**Background:**

The objective of this study was to validate the Impact of a Child with Congenital Anomalies on Parents (ICCAP) questionnaire. ICCAP was newly designed to assess the impact of giving birth to a child with severe anatomical congenital anomalies (CA) on parental quality of life as a result of early stress.

**Methods:**

At 6 weeks and 6 months after birth, mothers and fathers of 100 children with severe CA were asked to complete the ICCAP questionnaire and the SF36. The ICCAP questionnaire measures six domains: contact with caregivers, social network, partner relationship, state of mind, child acceptance, and fears and anxiety. Reliability (i.e. internal consistency and test-retest) and validity were tested and the ICCAP was compared to the SF-36.

**Results:**

Confirmatory factor analysis resulted in 6 six a priori constructed subscales covering different psychological and social domains of parental quality of life as a result of early stress. Reliability estimates (congeneric approach) ranged from .49 to .92. Positive correlations with SF-36 scales ranging from .34 to .77 confirmed congruent validity. Correlations between ICCAP subscales and children's biographic characteristics, primary CA, and medical care as well as parental biographic and demographic variables ranged from -.23 to .58 and thus indicated known-group validity of the instrument. Over time both mothers and fathers showed changes on subscales (Cohen's d varied from .07 to .49), while the test-retest reliability estimates varied from .42 to .91.

**Conclusion:**

The ICCAP is a reliable and valid instrument for clinical practice. It enables early signaling of parental quality of life as a result of early stress, and thus early intervention.

## Background

About 2–3% of newborn children exhibit major anatomical congenital anomalies (CA). Most of these are life-threatening unless surgically corrected [1]. Presentation may be isolated or as part of a spectrum of multiple congenital anomalies (MCA). Examples are intestinal atresia, abdominal wall defects, congenital diaphragmatic hernia (CDH), anorectal malformations and Hirschsprung's disease. Advances in surgery and peri-operative care have reduced mortality (apart from CDH) to approximately 10% [2]. This, however, has caused much more morbidity, with effects possibly extending into adulthood and placing a heavy burden on patients and parents, as well as on healthcare [3-5]. Earlier research by our group and others has shown that prenatal identification of CA can have considerable impact [6-9]. Therefore, it is presumed that postnatal impact of a child with CA may be even more striking and longer lasting.

Thinking about the serious consequences of (M)CA may induce a process of parental mourning. Abandoning expectations of a healthy child, parents must prepare themselves for raising a child being severely ill, either temporarily or life-long [10]. Children with CA face many problems, including multiple surgical interventions, long neonatal hospitalization, and often uncertainty about future quality of life. Delay in establishing the definitive picture of associated anomalies or the diagnosis of a syndromal pattern of malformation may even heighten parental insecurity, notably in the case of MCA.

While empirical research has evaluated parental burden experienced one year after the birth of a child with CA [11], little is known of parental adaptation during the first six months. The early stage is likely to be the most stressful period for parents. Many studies employed structured interviews and generic questionnaires at a later stage, not specifically geared to the particular situation of parents of a child with MCA [11-16]. An example of a generic questionnaire is the General Health Questionnaire [17,18]. The Perinatal Grief Scale [19,20] on the other hand is an example of a questionnaire developed for a specific condition, in this case grief. Nevertheless, none of the available instruments is specifically geared to the particular situation of parents with a malformed child. The more so because generic questionnaires lack specific domains of impact on parental burden, such as 'social support' and 'contact with caregivers'. In other words, parents will not recognize their specific situation in these generic questionnaires. Therefore, we constructed a new questionnaire designed to evaluate parental early stress and quality of life in the first 6 months after the birth of a child with (M)CA, the Impact of a Child with Congenital Anomalies on Parents (ICCAP) questionnaire. The intended use of the ICCAP is as an alert system to signal parents at risk of threatened quality of life.

We consider MCA patients and their parents to be a group that shares many characteristics. The ICCAP is specially targeted for this group because they are usually excluded from studies on outcome of neonatal intensive care. [21-23].

The aim of the study was the psychometric analysis of the ICCAP questionnaire as a potential tool for early intervention. It could be used in a clinical setting for early identification of parent-child couples who are most at risk for early stress.

## Methods

### Study population

The Erasmus MC-Sophia Children's Hospital is a university hospital with a 15-bedded tertiary pediatric surgical intensive care unit (PSICU) in which all surgical specialties except open-heart surgery are represented. A multidisciplinary treatment, support and evaluation team is available for the management of children with MCA and their parents.

Consecutive children with CA admitted to this PSICU from January 1999 to May 2001 were eligible for this study. Patients with meningomyelocele were excluded, because they already participated in the follow-up program of the multidisciplinary meningomyelocele team in our institution.

### Assessments

#### Instrument to be psychometrically tested: ICCAP

The ICCAP questionnaire was constructed as a self-report questionnaire for parents of children with any kind of CA. As an initial step we reviewed relevant questionnaires on psychological and social functioning to identify applicable domains for assessing early parental stress and quality of life. The General Health Questionnaire [17,18] and Perinatal Grief Scale [19,20] were most relevant in identifying divergent theoretical domains. Subsequently, items were formulated fitting these theoretical domains and representing aspects insufficiently covered by existing questionnaires.

In order to ensure adequate content validity, four experienced pediatric intensivists involved in the management of MCA patients independently identified indicators associated with MCA-related parental early stress. Indicators were classified into six domains: 1) contact with caregivers, signifying contact with medical and paramedical personnel and psychosocial support services, 2) social network, signifying contact with friends and family, 3) partner relationship, signifying the relationship with the co-parent of the child, 4) state of mind, signifying the state of mind parents find themselves in as a result of the birth of the child, 5) child acceptance, signifying the way the child can be accepted as a part of the family and 6) fears and anxiety, containing items describing fears, worries and anxiety about the immediate and long-term future of the child and the burden as experienced by both child and parent (see Table 1 for the contents of the items selected for each domain separately).

**Table 1:** Standardized factor loadings

		**6 weeks**		**6 months**	
**ICCAP dimensions**	**Item**	**Mothers **(n = 76)	**Fathers **(n = 71)	**Mothers **(n = 42)	**Fathers **(n = 41)

*Contact with caregivers*					
Doctors clearly explain things	17	.94	.77	.94	.99
I have good interaction with nurses	18	.74	.95	.70	.60
Doctors take enough time to listen to me	20	.74	.95	.94	.99
I am satisfied about my contacts with doctors	32	.74	.95	.86	.97

*Social network*					
My friends support me	02	.78	.82	.79	.90
My colleagues are understanding	11	.68	.54	.68	.49
People around me support me	19	.82	.90	.72	.95
My friends help me with practical things	21	.62	.81	.75	.67
I can share worries with my family	29	.76	.61	.59	.69
I can share worries with good friends	33	.91	.98	.84	.63

*Partner relationship*					
I feel my partner sympathizes with me	01	.94	.86	.89	.87
On important issues I agree with my partner	07	.98	.93	.96	.86
My partner is someone I can talk to	08	.98	.81	.93	.99
Generally I am happy with my partner	12	.96	.99	.94	.96
My relationship with my partner is good	14	.94	.99	.91	.93

*State of mind*					
I feel sad	03	.99	.89	.78	.86
I feel angry	16	.87	.89	.94	.74
I wonder whether I am to blame for my child's CA	22	.46	.70	.35	.42
I feel guilty	34	.61	.89	.57	.88

*Child acceptance*					
My child fits into my life	26	.87	.81	.99	.85
My child is welcome in our family as it is	31	.69	.79	.90	.89
I am happy with my child	35	.99	.87	.96	.84
I wish my child was never born	36	.70	.63	.80	.14

*Fears and anxiety*					
My child faces a difficult life	04	.62	.57	.70	.89
I expect my child will be able to function well	05	.85	.74	.96	.79
The CA is/are a heavy burden on my child	06	.76	.87	.73	.59
I wonder whether my child will ever be healthy	09	.80	.85	.61	.77
I am very anxious about all the tests on my child	10	.73	.46	.37	.59
My child is facing a difficult period	13	.86	.82	.86	.91
My child is the same as other children	15	.61	.84	.65	.70
I worry a great deal about my child's health	23	.79	.74	.87	.79
I doubt whether my child will be happy later	24	.72	.91	.90	.88
I fear about my child's expectations for the future	25	.85	.90	.90	.83
My child is handicapped	27	.72	.69	.68	.80
I feel I can't do enough for my child	28	.74	.59	.75	.74
My child will be able to have a normal life later	30	.80	.87	.76	.91

If required, items were rephrased to meet style criteria: unambiguous, concise, easily understandable and void of double negations. The original questionnaire comprised 82 items to be rated on a 5-point scale ranging from 1) strong agreement, 2) agreement, 3) disagreement, 4) strongly disagreement and 5) non applicable. Non applicable was scored when for instance contact with caregivers had not taken place (at 6 months). Positively phrased items were recoded as follows: 1 = 5, 2 = 4, 3 = 2, 4 = 1, and non-applicability was recoded as '3'. Thus, higher scores indicate a higher quality of life.

Subsequently, the items which were formulated fitting the theoretical domains parental stress and quality of life were allocated to these six domains: an item pool of multiple-choice questions was constructed into a prototype questionnaire. Then, the prototype questionnaire was reviewed for comprehensibility by a panel composed of two psychologists (one is a methodologist and one wrote a thesis on parental burden and grief [11]); clinicians; selected PSICU nursing staff of the unit; and selected parents. Interviewing an additional group of 20 parents, our social worker then evaluated the questionnaire for face validity and comprehension. A number of questions were modified in the light of advice and comments from these quarters.

#### Instrument for validation: Short Form 36

The SF-36 is a generic health status questionnaire [24-26]. It consists of 36 questions organized into 8 domains: 1) physical functioning; 2) social functioning; 3) role limitations because of physical health problems; 4) role limitations because of emotional problems; 5) general mental health; 6) vitality, 7) bodily pain and 8) general health. It also contains two summary measures: physical health (including domains 1, 3, 7 and 8) and mental health (including domains 2, 4, 5 and 6). Total scores are linearly transformed to range from 0 to 100, with higher scores indicating a better-perceived health status. A generic measure, the SF-36 has proven useful in surveys of general and specific populations.

### Background and medical variables

Table 2 lists the children's biographic characteristics, primary CA, and medical care as well as parental biographic and demographic variables used in the data analysis. Severity of disease was derived from the TISS (Therapeutic Intervention Scoring System) scores. The TISS is a well-known method of measuring factual intensity of nursing care in a hospital setting [27,28]. In our department TISS is used as a standard assessment score. (see Table 2)

**Table 2:** General characteristics of patients and parents

**Patients**	**N = 100**	
Female/male	41/59	
Gestational age (wks)	38 3/7*	(28 – 42 6/7)**
Birth weight (kg)	3.0*	(.75 – 4.51)**
***Primary anomalies***		
Abdominal wall defect	17	
Congenital diaphragmatic hernia	13	
Small intestinal anomaly	32	
Oesophageal atresia	15	
Anorectal malformation	4	
Hirschsprung's disease	5	
Miscellaneous	14	
Congenital anomalies (CA) per patient	2*	(1–7)**
***Medical care***		
Duration of first admission (days)	27.5*	(4–314)**
Total admission in first 6 months (days)	37*	(4–182)**
Period until complete diagnosis (days)	4*	(0–205)**
Medical appliances at discharge (number)	0*	(0–7)**
TISS ≥10 in first 6 months (days)	6*	(0–128)**

**Parents**		
Age mothers (yrs)	31*	(19–45)**
Age fathers (yrs)	33*	(23–50)**
Socioeconomic status		
low	22	
medium	55	
high	23	
CA in family	28	
Duration of parental relationship (yrs)	5*	(.50–20)**
Single parents (mothers)	4	
Sibs at time of birth (number)	1*	(0–5)**

* = median

** = range

TISS = Therapeutic Intervention Scoring System

### Design

This is a prospective, longitudinal study comprising two measurement moments: 6 weeks and 6 months after the birth of the child.

### Procedure

The Erasmus MC medical ethical review board approved the study. Parental written and signed informed consent was sought within the first week after an eligible child's birth. At both measurement moments parents were asked to complete two questionnaires: ICCAP and SF-36. Parent couples were explicitly instructed to complete the questionnaires independently. The questionnaires were either handed to parents on the ward or mailed to them after discharge of the child. Usually they were completed at home. When the questionnaires were not returned within two weeks, parents were telephoned once to remind them. Children's background and medical variables for assessing the child's condition and severity of disease were collected prospectively during admission and follow-up.

### Data analysis

A priori we postulated six theoretical domains. As the sample size was relatively small, we attempted to identify the dimensional structure for each separate empirically operationalised domain. To that end we applied the model generating strategy, after first having performed exploratory factor analysis to get an impression of the dimensionality of the data structure. This provided a far from clear structure. The model generating strategy, however, pointed to a confirmatory factor analysis solution. The main advantages of the latter approach are: 1) identifying and testing for model fit, 2) flexibility of estimating the factor intercorrelations, 3) enabling to fix parameters to certain values, 4) relaxing parameter values to be free and 5) enabling comparison of factor structures (in this study comparison of the two measurement moments).

Confirmatory factor analysis was applied for both measurement moments separately. The following measures of model performance were used: 1) χ^2^-tests for model fit in addition to the p-value corresponding to the χ^2^-value (preferably p-value >.05), 2) χ^2 ^divided by the degrees of freedom (preferably < 2.0), 3) comparative fit index (CFI preferably > .95), 4) Tucker-Lewis index (TLI preferably > .95), 5) Root Mean Square Error of Approximation (RMSEA preferably < .05) and 6) Weighted Root Mean Square Residual (WRMR preferably < 1.00). The standardized regression coefficient was used as a measure of relative importance for the individual variables. Variables used in the analysis were considered to be ordinal. Values were estimated using the weighted least square approach, applying a diagonal weight matrix with robust standard errors and mean- and variance-adjusted χ^2^-tests. For items to be selected, they had to load substantially (≥ .50) on preferably one factor. Also, all items loading onto the same factor had to be conceptually homogeneous.

For all subscales reliability was investigated using: 1) parallel estimates, 2) tau-equivalent estimates and 3) congeneric estimates [29,30]. If the squared loadings and the residual variances were equal, parallel estimates were allowed. If only the squared loadings are equal, tau-equivalence was allowed. If neither squared loadings nor the residual variances were equal, congeneric reliability was indicated. With congeneric measures, the reliability coefficient of the scale score equaled the summation of squared factor loadings for that scale, divided by the summation of squared factor loadings plus the summation of error variances [31,32]. For stability of the instrument test-retest reliabilities of the six empirically constructed scales were estimated.

To evaluate congruent validity of ICCAP, the two summary measures of the SF-36 were correlated with the domains of ICCAP using Spearman's rank order correlation coefficient (r_s_).

Likewise, to evaluate known-group validity, the background variables were correlated with the ICCAP domains. Confidence intervals (95%) were calculated for the correlation coefficients. No correlation was expected with background variables, except with those associated with severity of illness.

Concerning change over time (sensitivity to change and parental (dis)congruence): a measure for the probability of difference is presented, symbolized by the p-value (two-tailed), as well as a measure of the magnitude of change, symbolized by (Cohen's) d-measure of discrimination. A p-value below 0.05 indicates that the change is beyond chance level. In other words, in case of p < 0.05 the change is at least 1.96 × the standard error, signifying real change and not measurement instability. Cohen's d (rule of thumb: small = .20, moderate = .50 and high = .80) was used to indicate the magnitude of the differences between mothers and fathers at both measurement moments [33].

For correlations the rule of thumb for effect size provided by Cohen [33] was used: low = .10, moderate = .24 or high = .37.

Wilcoxon signed ranks test was used to determine significance for paired samples for ICCAP.

All statistical testing was performed at the .05 level of significance (two-tailed).

The software programs SPSS 14.0 for Windows and Mplus version 4.1 [34] were used.

## Results

### General characteristics

**Figure 1:**
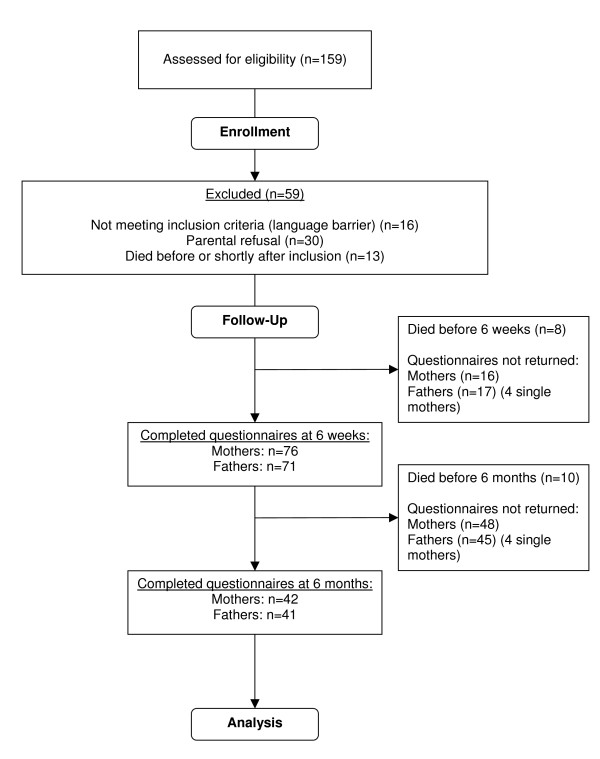
**Flowchart**.

From January 1999 to May 2001 a total of 159 eligible consecutive patients were admitted. Parents of 59 children did not participate for the following reasons: 13 children (8%) died before or shortly after study inclusion; in 16 cases (11%) parents lacked sufficient command of the Dutch language; and in 30 cases (19%) parents refused to participate for various reasons. Thus, parents of 100 children participated in the study, i.e. returned both questionnaires for at least one of the two measurement moments. This resulted in notably less than 100 repeated measurements as shown in Figure 1. Four children had single mothers.

Characteristics of children and parents are presented in Table 2. Diagnoses were equally distributed between the participating and non-participating groups, except for CDH, which was overrepresented in the non-participating group due to early deaths (10 out of 13 deaths).

Gestational age and birth weight were mostly within the lower range of normal. Forty percent of the children were still hospitalized at 6 weeks. Median duration of the first admission was 27.5 days; and median number of days on which TISS scores were ≥ 10 was 6. Most children (80%) were discharged before the age of 6 months.

### ICCAP structure determination

Confirmatory factor analysis was used to test the à priori assumption of a 6-factor model and expectations about which variables load onto which factors were tested likewise. The four factor analyses across time for both parents individually turned out to be similar within random fluctuation and resulted in a 6-factor solution with a total of 36 items contributing significantly to the empirical solutions. The remaining 46 items were deleted: some items overlapped; others did not fit any of the 6 factors; and some items with ambivalent phrasing were removed. The model performances for both parents across time appeared to be clinically satisfactory. Although the χ^2^-values turned out to be significant, yet the values of X^2 ^divided by the degrees of freedom were adequate (see Table 3). Also, CFI and TLI values were adequate, whereas RMSEA values were less satisfactory. The performance measure WRMR, most important performance measure for ordinal data, was clinically acceptable (see Table 3).

**Table 3:** Performance measures of model fit

	**6 weeks**		**6 months**	
	
	**Mothers**	**Fathers**	**Mothers**	**Fathers**
χ^2 I^	96.51	101.93	51.11	59.01
df ^II^	41	39	27	26
p ^III^	.001	.001	.003	.001
χ^2^/df	2.35	2.61	1.89	2.27
CFI ^IV^	.92	.92	.94	.94
TLI ^V^	.94	.94	.94	.94
RMSEA ^VI^	.13	.15	.15	.18
WRMR ^VII^	1.07	1.15	1.00	1.07

I χ^2^-test is a measure of model fit

II df indicates degrees of freedom of χ^2^-test

III p-values of the χ^2^-test

IV CFI = Comparative Fit Index

V TLI = Tucker – Lewis Index

VI RMSEA = Root Mean Square Error of Approximation

VII WRMR = Weighted Root Mean square Residual

Table 1 presents standardized factor loadings of the models identified for both parents for 6 weeks and 6 months respectively: most of these loadings exceeded the value of .70 (see Table 1).

Scores were linearly transformed to range from 0 to 100 with higher scores indicating a better-perceived status, analogous to the transformation as applied to SF-36 scales.

### Scale reliability

Congeneric scale reliability estimates were used, since factor loadings as well as the residual variances differed. Table 4 shows these reliability estimates for the six ICCAP domains at both measurement moments for both parents. Reliability estimates ranged from .49 to .92. On average reliability estimates did not show major differences across time for mothers or fathers. However, reliability estimates did differ for the different domains. The reliability estimate of the domain partner relationship was highest (mean:.87), with lower reliabilities of social network, fears and anxiety and state of mind (mean: around .58) (see Table 4). Test-retest reliabilities for the six scales turned out to be satisfactory, with values varying from .42 (contact with caregivers) to .91 (fears and anxiety).

**Table 4:** Reliabilities i.e. congeneric estimates of the six subscales of the ICCAP

		**6 weeks**		**6 months**	
**ICCAP dimensions**	**n° of items**	**Mothers**	**Fathers**	**Mothers**	**Fathers**

Contact with caregivers	4	.63	.83	.75	.82
Social network	6	.59	.63	.54	.55
Partner relationship	5	.92	.84	.86	.85
State of mind	4	.58	.72	.49	.56
Child acceptance	4	.68	.60	.84	.56
Fears and anxiety	13	.58	.59	.58	.63

### Congruent validity

Moderate to high correlations, ranging from .34 to .77, were found between state of mind and the SF-36 summary measures for both mothers and fathers, at 6 weeks and, more outspoken, at 6 months. In addition, fears and anxiety for both parents appeared to be substantially related to the SF-36 mental component scale, with correlations ranging from .30 to .43.

### Known-group validity

Moderate to high correlations with mostly child-related background variables were found for fears and anxiety for both parents at 6 weeks and 6 months, with coefficients ranging from -.30 to -.58 and to a lesser extent for state of mind for mothers at 6 weeks, ranging from -.23 to -.41. These significantly correlated background variables were mainly related to severity of illness of the child, and included duration of admission and number of medical appliances at discharge.

A significant negative correlation (r_s_) was found between parental age and partner relationship (-.35 to -.45) at 6 months for both parents. Acceptance of the child at 6 months turned out to be negatively correlated with duration of parental relationship for both parents (-.35 and -.40). For fathers, contact with caregivers showed significant positive correlations with gestational age at both measurement moments (.29 for 6 weeks and .53 for 6 months).

### Sensitivity to change

Over time ICCAP showed change for mothers and fathers, mainly on the parental relationship domain, with Cohen's d of -.47 and -.49, respectively (see Additional file 1). Significant positive change over time was found for fears and anxiety, in paired measurements, for both parents (mothers: Wilcoxon test, z = -1.99, p = .04, n = 34, fathers: z = -2.37, p = .02, n = 34). Negative change was found for partner relationship (mothers: z = 1.90, p = .03, n = 33, fathers: z = 1.92, p = .03, n = 34) (see Additional file 1).

### Parental (dis)congruence

Additional file 1 shows comparable ICCAP scores for fathers and mothers, indicated by low Cohen's d (< .20), both at 6 weeks and at 6 months, with the exception of state of mind (d = .27 and .37, respectively). The higher levels of agreement for both parents were reached on acceptance of the child and partner relationship, with lower agreement between parental levels on fears and anxiety, contact with caregivers and social network.

At 6 weeks paired measurements showed significant differences between parents for two domains: contact with caregivers (Wilcoxon test: z = 1.55, p = .04, n = 65) and fears and anxiety (z = -2.01 p = .04, n = 69), and at 6 months for state of mind only (z = -2.53, p = .04, n = 41).

Both fathers and mothers clearly perceived lower quality of life than the norm group, particularly on the SF-36 mental component scale (see Additional file 1). At 6 weeks fathers of children with CA had higher scores than the norm group on the physical component scale although at 6 months scores had decreased to slightly below the norm.

Mothers perceived lower quality of life than did fathers, both at 6 weeks and 6 months. The physical component scale shows the greatest discrepancy between parents at 6 weeks.

## Discussion

The purpose of this study was to validate a new questionnaire designed to measure the impact of early stress on quality of life of parents confronted with a newborn baby showing severe birth defects. From confirmatory factor analysis it appeared feasible to reduce a first 82-item version to 36 items in a 6-domain model. The number of items per domain range from 4 to 6, except for fears and anxiety, which contains 13 items. We felt this domain is best geared to detect impact in this specific group of parents, and may therefore carry heavier weight.

In this study we established three kinds of validity, i.e. congruent validity, known-group validity, and sensitivity to change. First, concerning congruent validity, the ICCAP domain state of mind positively correlated with the SF-36 mental and physical component scales at both measurement moments and for both parents. The domain fears and anxiety similarly correlated with the mental component scale for both parents. For the other ICCAP domains correlations are less outspoken, implying that ICCAP and SF-36 measure different aspects of parental functioning. The theoretical and empirical constructs clearly differ. ICCAP aims to measure quality of life as a result of parental stress and is more differentiated than SF-36, whereas the latter aims to measure general quality of life. In conclusion, ICCAP gives additional specific information when used next to the SF-36.

Second, known-group validity is supported by the fact that severity-of-illness variables showed considerable correlations with state of mind and fears and anxiety. These correlations are consistent at both measurement moments with a slight decrease in magnitude at 6 months.

High parental age and longer duration of parental relationship were risk factors for parental relationship and child acceptance, respectively, for both parents.

Concerning sensitivity to change as a third measure of validation, the level of fears and anxiety felt for the child and its future appeared to decrease significantly over time for both parents. Two possible explanations present themselves. On the one hand, parents may have gained better understanding of what to expect in the future. On the other hand, the acute severity of disease and the child's discomfort will usually have abated over time. This sensitivity to change in ICCAP makes the instrument useful in a clinical setting, the more so as it could alert to changes in risk for early stress.

We also looked at parental (dis)congruence. On most domains there was parental congruence, increasing over time. This may be partly due to maternal physical recovery. Only on state of mind we observed parental discongruence increasing over time. Parental discongruence in parents of the same child on contact with caregivers and fears and anxiety disappears over time. This is replaced by discongruence in state of mind. Discrepancies in reported impact by parents of the same child might be an indicator of impact, suggesting lack of communication, unequal burden and other possible disturbances in parental relationship.

ICCAP fits clinical practice very well, especially since the questions are easy to understand and completion takes only 10 minutes. It may also serve as a screening tool to identify parents in need of support from a psychologist or a social worker. Furthermore, we are in the process of developing a user-manual, presenting norms of larger CA population samples.

Our study has a limitation in that data assessed at 6 months are based on a relatively small sample size (n = 41–42). Larger sample sizes are needed to show whether these correlations might be of clinical significance. Further data collection and analysis of data are, however, part of ongoing investigation in our institute.

## Conclusion

The ICCAP is a reliable and valid instrument for clinical practice. It enables early signaling of parental quality of life as a result of early stress. After cross validation of ICCAP in a new, larger, study group we will be able to determine ICCAP cut-off scores that signal high risk for early stress. Tailored interventions to ease the parental adaptation process can thus be evaluated.

## Abbreviations

CA: Congenital Anomaly; MCA: Multiple Congenital Anomalies; CDH: Congenital Diaphragmatic Hernia; ICCAP: Impact of a Child with Congenital Anomalies on Parents questionnaire; PSICU: Pediatric Surgical Intensive Care Unit; TISS: Therapeutic Intervention Scoring System; CFI: Comparative Fit Index; TL: Tucker-Lewis index; RMSEA: Root Mean Square Error of Approximation; WRMR: Weighted Root Mean square Residual; r_s_: Spearman's rank order correlation coefficient.

## Competing interests

The authors declare that they have no competing interests.

## Authors' contributions

All authors made intellectual contributions and contributed to the writing of the manuscript. PM and SJG conceived of the study instrument, collected the data for this study, contributed to the data analysis, the interpretation of the data and drafted the manuscript. MD and HJD contributed to the statistical analysis, the interpretation of the data and helped to draft the manuscript. HMK and DT participated in the design and contributed to critical revision of the manuscript. All authors read and approved the final version of the manuscript.

## Supplementary Material

Additional file 1 **Table 5**. SF-36 and ICCAP distinguished by parent across time. Click here for file
