# Supplementary material for: Impact of a child with congenital anomalies on parents (ICCAP) questionnaire; a psychometric analysis
Source: Health Qual Life Outcomes. 2008 Nov 23;6:102. doi: 10.1186/1477-7525-6-102 (PMC2607266; doi:10.1186/1477-7525-6-102)
Supplement: Additional file 1 — Table 5. SF-36 and ICCAP distinguished by parent across time. [file 1477-7525-6-102-S1.doc]

**Table 5 -** SF-36 and ICCAP distinguished by parent across time

|  | **6 weeks** | | | | | | | **6 months** | | | | | | | **6 weeks – 6 months** | |
| --- | --- | --- | --- | --- | --- | --- | --- | --- | --- | --- | --- | --- | --- | --- | --- | --- |
|  | **Mothers** | | | **Fathers** | | |  | **Mothers** | | | **Fathers** | | |  | **Mothers** | **Fathers** |
| **ICCAP** | $\bar{\text{x}}$ | sd | n | $\bar{\text{x}}$ | sd | n | d* | $\bar{\text{x}}$ | sd | n | $\bar{\text{x}}$ | sd | n | d* | d* | d* |
| Contact with caregivers | 78.63 | 15.94 | 72 | 75.57 | 21.15 | 68 | -.16 | 75.39 | 15.81 | 36 | 76.97 | 15.39 | 38 | .10 | -.20 | .07 |
| Social network | 76.39 | 16.38 | 73 | 73.75 | 19.71 | 69 | -.15 | 73.30 | 14.99 | 38 | 71.36 | 17.53 | 38 | -.12 | -.19 | -.13 |
| Partner relationship | 89.88 | 13.53 | 72 | 89.58 | 13.27 | 71 | -.02 | 83.00 | 16.33 | 40 | 82.11 | 17.92 | 41 | -.05 | -.47 | -.49 |
| State of mind | 68.15 | 22.49 | 75 | 74.29 | 23.86 | 70 | .27 | 71.76 | 21.08 | 42 | 79.20 | 18.79 | 41 | .37 | .16 | .22 |
| Child acceptance | 92.00 | 12.65 | 76 | 90.77 | 11.80 | 71 | -.10 | 89.35 | 15.59 | 42 | 88.96 | 13.66 | 40 | -.03 | -.19 | -.14 |
| Fears and anxiety | 61.74 | 22.98 | 74 | 64.37 | 21.44 | 70 | .12 | 66.70 | 23.06 | 42 | 67.08 | 22.99 | 41 | .02 | .22 | .12 |
| **SF36** |  |  |  |  |  |  |  |  |  |  |  |  |  |  |  |  |
| PCS ($\bar{\text{x}}$=83.4; sd=24.1)^‡^ | 76.67 | 19.90 | 68 | 85.24 | 14.81 | 64 | .49 | 76.69 | 20.59 | 79 | 83.18 | 22.03 | 71 | .30 | -.00 | -.11 |
| MCS ($\bar{\text{x}}$=81.4; sd=21.3)^‡^ | 62.68 | 20.76 | 68 | 67.02 | 23.74 | 64 | .20 | 64.56 | 21.93 | 79 | 72.27 | 23.99 | 71 | .34 | .09 | .22 |

$\bar{\text{x}}$ = Mean

sd = Standard deviation

* Cohen’s d was used where the standard deviation was pooled

PCS = Physical Component Scale

MCS = Mental Component Scale

‡ Population norms: n=221, age 25-34 (24)
